# Supplementary material for: Heat adaptation of phage T7 under an extended genetic code
Source: Virus Evol. 2021 Dec 1;7(2):veab100. doi: 10.1093/ve/veab100 (PMC8923235; doi:10.1093/ve/veab100)
Supplement: veab100_Supp [file veab100_supp.zip › Sequencing_Analysis.zip!/Sequencing_Analysis/A3/marginal.html]

BRESEQ :: Marginal Predictions


|  |  |
| --- | --- |
|  | ***breseq***  version 0.30.1    mutation predictions \| marginal predictions \| summary statistics \| genome diff \| command line log |

| Marginal read alignment evidence | | | | | | | | | | | |
| --- | --- | --- | --- | --- | --- | --- | --- | --- | --- | --- | --- |
|  | seq id | position | | ref | new | freq | score (cons/poly) | reads | annotation | genes | product |
| \* | NC\_001604.1 | 35,254 | 0 | A | G | 59.1% | 943.5 / inf | 1648 | intergenic (–/–) | *–/–* | –/– |
| \* | NC\_001604.1 | 29,691 | 0 | A | G | 57.8% | 795.6 / inf | 1484 | intergenic (–/–) | *–/–* | –/– |
| \* | NC\_001604.1 | 30,576 | 0 | C | T | 51.9% | 56.2 / inf | 1629 | intergenic (–/–) | *–/–* | –/– |
| \* | NC\_001604.1 | 27,827 | 0 | G | C | 49.5% | 141.3 / inf | 1842 | intergenic (–/–) | *–/–* | –/– |
| \* | NC\_001604.1 | 167 | 0 | T | A | 48.9% | 41.8 / inf | 1414 | intergenic (–/–) | *–/–* | –/– |
| \* | NC\_001604.1 | 33,023 | 0 | A | G | 38.2% | 1351.7 / inf | 2086 | intergenic (–/–) | *–/–* | –/– |
| \* | NC\_001604.1 | 1,242 | 2 | . | A | 27.9% | 2588.4 / inf | 1847 | intergenic (–/–) | *–/–* | –/– |
| \* | NC\_001604.1 | 1,242 | 5 | . | A | 27.9% | 2569.9 / inf | 1847 | intergenic (–/–) | *–/–* | –/– |
| \* | NC\_001604.1 | 1,242 | 6 | . | A | 27.9% | 2591.7 / inf | 1847 | intergenic (–/–) | *–/–* | –/– |
| \* | NC\_001604.1 | 1,242 | 7 | . | G | 27.9% | 3068.4 / inf | 1847 | intergenic (–/–) | *–/–* | –/– |
| \* | NC\_001604.1 | 1,242 | 1 | . | G | 27.8% | 3018.8 / inf | 1847 | intergenic (–/–) | *–/–* | –/– |
| \* | NC\_001604.1 | 1,242 | 4 | . | G | 27.7% | 3089.0 / inf | 1847 | intergenic (–/–) | *–/–* | –/– |
| \* | NC\_001604.1 | 1,242 | 3 | . | G | 27.6% | 3101.7 / inf | 1847 | intergenic (–/–) | *–/–* | –/– |
| \* | NC\_001604.1 | 1,242 | 8 | . | T | 27.6% | 2651.2 / inf | 1847 | intergenic (–/–) | *–/–* | –/– |
| \* | NC\_001604.1 | 1,242 | 9 | . | C | 27.5% | 3144.0 / inf | 1847 | intergenic (–/–) | *–/–* | –/– |
| \* | NC\_001604.1 | 19,625 | 0 | G | T | 20.0% | 3611.2 / inf | 1523 | intergenic (–/–) | *–/–* | –/– |

| Marginal new junction evidence (lowest skew 10 of 21 shown) | | | | | | | | | | | |
| --- | --- | --- | --- | --- | --- | --- | --- | --- | --- | --- | --- |
|  | | seq id | position | reads (cov) | reads (cov) | score | skew | freq | annotation | gene | product |
| \* | ? | NC\_001604.1 | = 13354 | 265 (0.150) | 339 (0.200) | 173/594 | 4.9 | 53.9% | intergenic (–/–) | *–/–* | –/– |
|  | ? | NC\_001604.1 | 14356 = | 317 (0.180) |  |  |  |  | intergenic (–/–) | *–/–* | –/– |
| \* | ? | NC\_001604.1 | = 13455 | 69 (0.040) | 234 (0.140) | 134/586 | 8.0 | 71.9% | intergenic (–/–) | *–/–* | –/– |
|  | ? | NC\_001604.1 | 14331 = | 116 (0.070) |  |  |  |  | intergenic (–/–) | *–/–* | –/– |
| \* | ? | NC\_001604.1 | = 1256 | 0 (0.000) | 119 (0.070) | 65/574 | 18.1 | 100% | intergenic (–/–) | *–/–* | –/– |
|  | ? | NC\_001604.1 | 2737 = | 0 (0.000) |  |  |  |  | intergenic (–/–) | *–/–* | –/– |
| \* | ? | NC\_001604.1 | = 7781 | 1662 (0.950) | 68 (0.040) | 53/558 | 20.4 | 4.1% | intergenic (–/–) | *–/–* | –/– |
|  | ? | NC\_001604.1 | 7899 = | 1673 (1.030) |  |  |  |  | intergenic (–/–) | *–/–* | –/– |
| \* | ? | NC\_001604.1 | = 13578 | 5 (0.000) | 145 (0.090) | 52/584 | 21.5 | 54.1% | intergenic (–/–) | *–/–* | –/– |
|  | ? | NC\_001604.1 | 14348 = | 241 (0.140) |  |  |  |  | intergenic (–/–) | *–/–* | –/– |
| \* | ? | NC\_001604.1 | = 13586 | 0 (0.000) | 152 (0.090) | 52/592 | 21.8 | 51.1% | intergenic (–/–) | *–/–* | –/– |
|  | ? | NC\_001604.1 | 14356 = | 291 (0.170) |  |  |  |  | intergenic (–/–) | *–/–* | –/– |
| \* | ? | NC\_001604.1 | = 281 | 1741 (1.120) | 373 (0.220) +TAAAGACCAGACC | 41/508 | 22.1 | 16.7% | intergenic (–/–) | *–/–* | –/– |
|  | ? | NC\_001604.1 | 282 = | 1933 (1.110) |  |  |  |  | intergenic (–/–) | *–/–* | –/– |
| \* | ? | NC\_001604.1 | = 7530 | 2179 (1.250) | 29 (0.020) | 24/580 | 31.0 | 1.4% | intergenic (–/–) | *–/–* | –/– |
|  | ? | NC\_001604.1 | 8037 = | 2008 (1.190) |  |  |  |  | intergenic (–/–) | *–/–* | –/– |
| \* | ? | NC\_001604.1 | = 13568 | 14 (0.010) | 134 (0.080) | 16/586 | 35.3 | 58.7% | intergenic (–/–) | *–/–* | –/– |
|  | ? | NC\_001604.1 | 14339 = | 175 (0.100) |  |  |  |  | intergenic (–/–) | *–/–* | –/– |
| \* | ? | NC\_001604.1 | = 7613 | 2216 (1.270) | 19 (0.010) | 11/586 | 38.5 | 0.93% | intergenic (–/–) | *–/–* | –/– |
|  | ? | NC\_001604.1 | 7912 = | 1900 (1.110) |  |  |  |  | intergenic (–/–) | *–/–* | –/– |
